# Catheter Associated Blood Stream Infections in Patients Receiving Parenteral Nutrition: A Prospective Study of 850 Patients

**DOI:** 10.4021/jocmr1032w

**Published:** 2013-01-11

**Authors:** Ann O'Connor, Ann M Hanly, Eamonn Francis, Noreen Keane, Deborah A McNamara

**Affiliations:** a Department of Surgery, Beaumont Hospital, Dublin 9, Ireland; b Department of Surgery, St James Hospital, Dublin 8, Ireland; c Department of Colorectal Surgery, Beaumont Hospital, Dublin 9, Ireland

**Keywords:** Total Parenteral Nutrition, Catheter related blood stream infection, Central venous catheterisation

## Abstract

**Background:**

Data was prospectively collected on 850 consecutive patients undergoing central venous catheterisation (CVC) to receive total parenteral nutrition (TPN) in a major university teaching hospital over a 46 months period.

**Methods:**

Data included information about CVC insertion and clinical outcomes, most notably, suspected catheter-related blood stream infections (CRBSI).

**Results:**

The internal jugular vein was the most common site (n = 882, 68%), followed by the subclavian vein (n = 344, 24.6%) and femoral vein (n = 95, 6.5%). The CRBSI rate per 100 line feeding days was 0.93% in patients cared for in a non ICU setting versus 1.98% for ICU managed patients. The mean number of line days preceding a pyrexial spike was 13.1. CRBSI was commonest in patients with femoral lines (n = 21/95, 22.1%), especially those cared for in a non-ICU setting (29.6% versus 14.5% for those in the ICU group). Preference should be given to internal jugular or subclavian-sited CVCs in ICU and non-ICU patients to reduce the risk of CRBSI. If femoral catheterisation is unavoidable, strict attention to aseptic technique is mandatory.

**Conclusion:**

The aim of this study was to investigate the rate and pattern of CRBSI and to recommend changes in protocol, technique and equipment as deemed necessary from these findings.

## Introduction

Parenteral nutritional support involves the intravenous provision of calories, amino acids, electrolytes, vitamins, minerals, trace elements and fluids to meet the metabolic needs of critical illness or in patients for whom enteral nutrition is unsuitable or insufficient. Central venous catheterisation (CVC) is the route of choice for total parenteral nutrition (TPN) as its osmotic load is not well tolerated by peripheral veins. Access is most commonly via subclavian, internal jugular or femoral veins with tunnelled lines being used in less acute or more long-term situations. As well as the risk associated with their underlying condition, patients who receive parenteral nutritional support are at risk of catheter-related blood stream infections (CRBSI), adverse metabolic effects, and complications related to venous access. We prospectively collected data concerning a consecutive series of 850 patients receiving TPN via CVC over a 4 year period. The aim of this study was to investigate the rate and pattern of CRBSI in a major university hospital and to recommend changes in protocol, technique and equipment as deemed necessary from these findings.

## Material and Methods

An epidemiological prospective study was conducted over a 46 months period between March 2006 and December 2009 in a large university teaching hospital in Dublin. All data was collected by a single observer, a parenteral nutrition nurse-specialist. All patients receiving TPN in an ICU and non-ICU setting were included. Informed consent was obtained from each patient prior to catherisation.

All CVCs were inserted using standard strict sterile precautions consistent with the Healthcare Infection Control Practices Advisory Committee guidelines [1]. CVCs were sited by an anaesthetic consultant or registrar in either an ICU or operating theatre setting under strict aseptic conditions. A nursing protocol for care of central venous lines was in place and the nurse specialist inspected lines for compliance and for evidence of contamination. CVCs were removed if there was a suspicion of infection, when the catheter was no longer required and routinely after 14 days. As per standard practice when CRBSI was suspected the catheter was removed and the tip cultured for micro-organisms, blood cultures were sent and infection elsewhere was ruled out via a standard septic screen. Catheter related sepsis was defined as per the Centre for Disease Control (CDC) guidelines [2]. CRBSI was documented when other potential sources of infection were excluded and where a culture of the catheter tip demonstrated substantial colonies of an organism identical to those found in the bloodstream.

The CVC insertion site, number of previous CVC insertions, type of catheter, duration of central venous catheterisation and events of CRBSI were recorded. The procedural setting and grade of operator were recorded. Clinical septicaemia was recorded in the circumstance of systemic inflammatory response syndrome and associated pathogenic organisms identified in the blood. Those patients with clinical septicaemia and identical organisms cultured from their blood and catheter tip were considered to have a CRBSI.

Results were analysed using Predictive Analytics Software (PASW 18·0·2, SPSS Inc., Chicago, USA). The effect of CVC type, site and procedural setting on CRBSI rates was expressed as an odds ratio (OR) with 95% confidence intervals (CI). All tests of significance were 2-tailed. Results were considered significant where an OR did not equal 1, the 95% CI did not cross 1 and the p value was less than 0.05.

## Results

A total of 850 patients underwent central venous catheterisation for total parenteral nutrition between March 2006 and December 2009. A total of 1,321 CVCs were sited; 529 in ICU-managed patients and 792 in non-ICU patients, 87 were inserted by a consultant anaesthetist and 1,234 by an anaesthetic registrar. Consultant-inserted CVCs were more likely to result in clinical sepsis but this did not reach significance (14.9% versus 11%; P = 0.225).

The percentage of CVC cases meeting bacteriological criteria for CRBSI and demonstrating clinical septicaemia in the ICU and non-ICU group was 11.2% and 12.1% respectively (P = 0.602). Access was via an internal jugular vein in 882 cases (68%), the subclavian vein in 344 cases (24.6%) and femoral vein in 95 cases (6.5%) (Table 1). CVC-attributed clinical sepsis was highest in patients with femoral vein catheters, occurring in 21 of 95 cases (22.1%; 95% CI 1.33 to 3.98, P < 0.003). CRBSI was more likely in jugular-sited lines (11.7%; 95% CI 0.68 to 1.43, P = 0.92) compared to subclavian lines (9.0%; 95% CI 0.44 to 1.05, P = 0.08). Triple lumen catheters were utilised most frequently (69%), followed by double (20%) and quadra (11%) lumen catheters. CRBSI was more likely with 3 or 4 lumen CVCs than a 2 lumen CVC which had a CRBSI rate similar to that of tunnelled lines (Table 2).

**Table 1:** Patients Clinical Data.

	Non ICU	ICU
Number of Patients	322	528
Number of CVC	529	792
% Rate of TPN related line sepsis	11.2%	12.1%
Total number of line feeding days	3,495	8,808
Sepsis per 100 line feeding days	1.98%	0.93%
Mean number of days post line insertion pyrexia	12.79	9.26

**Table 2:** Central Venous Catheterisation Types and Sites.

	Sepsis Rate%	OR	P-value	Upper CI	Lower CI
Line Type					
Dual-lumen	8.3	0.69	0.310	0.34	1.36
Triple-lumen	13.8	1.30	0.148	0.90	1.86
Quadra-lumen	14.3	1.85	0.078	0.94	3.59
Tunnelled line	7.8	0.55	0.062	0.28	1.08
Line Site					
Femoral*	22.1	2.31	0.003	1.33	3.98
Jugular	11.7	0.98	0.928	0.68	1.43
Subclavian	9.0	0.68	0.079	0.44	1.05

The total number of line feeding days was 4,753 days, 3,495 for the ICU population and 1,258 for the non-ICU population. Sepsis per 100 line feeding days was 1.45% overall, 0.93% in the non-ICU population compared to 1.98% in the ICU population.

## Discussion

CVC-related bloodstream infection is suggested if a primary bloodstream infection develops in a patient who had a CVC in-situ within the preceding 48-hour period. Peripheral blood cultures positive for staphylococcus aureus, coagulase-negative staphylococci or candida species in the absence of other identifiable infective sources should increase the suspicion for catheter-related blood stream infection (CRBSI) [3].

As per CDC guidelines, CRBSI is confirmed when synchronous peripheral blood stream and central venous catheter tip cultures demonstrate substantial colonies of an identical organism.

Patients receiving parenteral nutrition via CVC are at increased risk of acquiring a bloodstream infection, compared to those with CVCs for other indications [4]. Among patients receiving parenteral nutrition, factors independently associated with bloodstream infection include poor patient hygiene, emergency central venous catheterisation, and, to a lesser extent, the severity of illness and duration of catheterisation [5].

A multicentre randomised control trial demonstrated a significant increase in overall infection (20 versus 3.7 per 1,000 catheter-days), clinical sepsis (4.5 versus 1.2 per 1,000 catheter-days), and thrombotic complications in femoral versus subclavian CVCs. This study concurs, showing a significant increase in line sepsis in patients with femoral vein catheters compared to other sites. Subclavian vein-sited lines were the least likely to result in CRBSI when compared to other sites. The subclavian vein should be the site of choice for CVCs followed by the internal jugular vein, avoiding use of the femoral vein where possible.

The risk of infection with central venous catheters increases with the duration of use [6]. However, a defined time course for routine change of catheters has not been conclusively established [7]. Vigilant clinical evaluation and assessment of the catheter site should be performed at least every other day. We demonstrated that the mean number of days preceding a pyrexial spike with a CVC in-situ was 13.1. Our current hospital policy of routinely changing CVCs is 14 days.

Several studies have shown multi-lumen catheters to have an increased rate of CRBSI when compared to single lumen catheters. There was no statistical difference in the rate of CRBSI in the different groups in our study although there was a trend toward an increased contamination rate in the higher lumen groups. Double lumen catheters had the lowest rate of CRBSI, a rate that was similar to that of tunnelled lines. Current evidence does not support the routine tunnelling of short-term catheters especially in an acute setting therefore these results suggest double-lumen catheters should be considered an appropriate choice in the setting of TPN use.

Optimum hand hygiene and maximal barrier precautions reduce the rate of CRBSI [8], however a 2006 report of intensive care units in 10 academic tertiary-care hospitals note a less than 30% compliance rate [9]. This suggests that compliance with strict sterile technique may somewhat ameliorate these and other results and reduce the rate of CRBSIs seen in hospitals.

### Conclusions

In conclusion, our 4 year experience demonstrates that the location and number of lumens are important considerations when planning a CVC placement for TPN. Despite advances in sterile technique, management of lines and treatment of sepsis, CRBSI is still a significant cause of morbidity and potential mortality in critical care patients. Resulting from our prospective review, we recommend the routine change of CVC’s is 12 days.
